# Temporal and Tissue Specific Regulation of RP-Associated Splicing Factor Genes *PRPF3, PRPF31* and *PRPC8—Implications in the Pathogenesis of RP*

**DOI:** 10.1371/journal.pone.0015860

**Published:** 2011-01-19

**Authors:** Huibi Cao, Jing Wu, Simon Lam, Rongqi Duan, Catherine Newnham, Robert S. Molday, John J. Graziotto, Eric A. Pierce, Jim Hu

**Affiliations:** 1 Physiology and Experimental Medicine Program, University of Toronto, Toronto, Canada; 2 Department of Laboratory Medicine and Pathobiology, University of Toronto, Toronto, Canada; 3 Department of Paediatrics, University of Toronto, Toronto, Canada; 4 Department of Biochemistry and Molecular Biology, University of British Columbia, Vancouver, Canada; 5 F.M. Kirby Center for Molecular Ophthalmology, Scheie Eye Institute, University of Pennsylvania School of Medicine, Philadelphia, Pennsylvania, United States of America; King's College London, United Kingdom

## Abstract

**Background:**

Genetic mutations in several ubiquitously expressed RNA splicing genes such as *PRPF3*, *PRP31* and *PRPC8*, have been found to cause retina-specific diseases in humans. To understand this intriguing phenomenon, most studies have been focused on testing two major hypotheses. One hypothesis assumes that these mutations interrupt retina-specific interactions that are important for RNA splicing, implying that there are specific components in the retina interacting with these splicing factors. The second hypothesis suggests that these mutations have only a mild effect on the protein function and thus affect only the metabolically highly active cells such as retinal photoreceptors.

**Methodology/Principal Findings:**

We examined the second hypothesis using the *PRPF3* gene as an example. We analyzed the spatial and temporal expression of the *PRPF3* gene in mice and found that it is highly expressed in retinal cells relative to other tissues and its expression is developmentally regulated. In addition, we also found that *PRP31* and *PRPC8* as well as *sn*RNAs are highly expressed in retinal cells.

**Conclusions/Significance:**

Our data suggest that the retina requires a relatively high level of RNA splicing activity for optimal tissue-specific physiological function. Because the RP18 mutation has neither a debilitating nor acute effect on protein function, we suggest that retinal degeneration is the accumulative effect of decades of suboptimal RNA splicing due to the mildly impaired protein.

## Introduction

The removal of introns from nascent precursor messenger RNA (pre-mRNA) transcripts is catalyzed by the spliceosome, a large and dynamic RNA-protein complex in the cell nucleus [1]. Spliceosome assembly requires sequential formation of a number of stable macromolecular intermediate complexes, each containing small nuclear ribonucleoproteins (snRNPs), a pre-mRNA and non-snRNP-associated proteins. Each snRNP contains one (U1, U2, or U5) or two (U4 and U6) uridine-rich, small nuclear RNAs (snRNAs) associated with a set of proteins that are either common to all snRNPs (Sm and LSm proteins) or specific to a given particle [2], [3]. Hprp3p, the product of the human *PRPF3* gene, is one of the proteins specifically associated with the U4/U6 snRNP complex [4], [5]. Hprp3p is highly conserved from yeast to human, particularly at its carboxyl terminus. This region is essential for Hprp3p function by engaging in a network of interactions with snRNAs and proteins that may stabilize intermediate complexes required for the assembly of the spliceosome [6]. In particular, the Hprp3p C-terminus is both necessary and sufficient for binding to Hprp4p and U4/U6 snRNA. Additionally, it contacts the U4/U6 snRNA recycling factor p110, SPF30 (one of the U2 snRNP proteins) as well as two U5 snRNP associated proteins, Hprp6 and Hsnu66 [7], [8], [9]. Moreover, substitution of threonine-494 for methionine at the C terminus of Hprp3p is involved in the etiology of autosomal-dominant retinitis pigmentosa type 18 (RP18) [10].

Retinitis pigmentosa (RP) is one of the most common forms of retinal degenerative diseases affecting 1 in 4000 people worldwide [11]. RP defines a heterogeneous group of retinal degenerative diseases characterized by the gradual structural and functional disruption of photoreceptor cells, retinal pigment epithelium and choroid layers [12]. Affected individuals first experience defective dark adaptation, followed by constriction of the peripheral visual field and, in the later stages of the disease, loss of central vision. Approximately 50 percent of diagnosed individuals have no known family history of the disease. These cases are called sporadic or RP simplex [13], [14]. The remaining 50 percent of affected individuals have a known family history. Familial RP displays all three modes of Mendelian inheritance: autosomal dominant (adRP), autosomal recessive (arRP) and X-linked (xlRP) [14], [15]. The RP-causing genes are involved in a variety of cellular functions, including the phototransduction cascade, vitamin A metabolism, cytoskeletal structure, RNA splicing, cell-cell interaction, trafficking of intracellular proteins, phagocytosis, pH regulation and others [11]. The genetic complexity of RP has been highlighted by the discovery of RP mutations not only in *PRPF3* but also in other pre-mRNA splicing genes (*PRPF31* for RP11 and *PRPC8* for RP13) [10], [16], [17], [18]; these splicing genes function not only in the retina but also throughout the human body [12], [17], [18], [19]. The discovery of adRP mutations in these genes clearly indicates the importance of having two wild-type copies of these genes for photoreceptor survival and function. Currently, the molecular mechanisms by which ubiquitous pre-mRNA splicing genes confer a tissue-specific pathogenesis are not clear [20]. However, several theoretical models have been proposed for testing the underlying mechanisms such as haploinsufficiency, splicing rate-limitation and disruption of interactions of splicing factors with photoreceptor-specific cofactor(s). Here we hypothesize that the retina demands high levels of splicing components and a slightly reduced level of splicing activity may have a cumulative and more serious consequence in photoreceptor cells than in other cell types because photoreceptors are highly metabolically active [21], [22].

In this paper, we examined the temporal and spatial expression of splicing factors that are directly involved in RNA splicing. We found that the expression of only RP-associated genes (*PRPF3, PRP31* and *PRP8*), which encode splicing factors for the U4/U6/U5 tri-snRNP, is significantly higher in the retina than in other tissues of adult mice while the expression of genes encoding non-RP splicing factors is not increased. We also found that levels of snRNAs, including U1, U2, U4, U5, and U6, are more abundant in retina than in other tissues. These results indicate that the postnatal retina requires higher steady-state basal levels of splicing components than do other metabolically active tissues.

## Results

### Temporal and spatial expression of *PRPF3* mRNA and protein in mice

**Figure 1:**
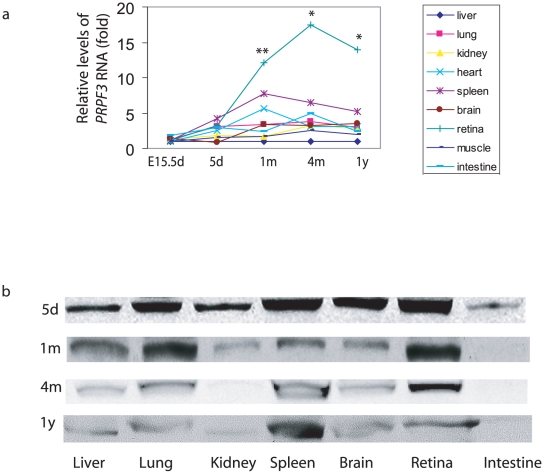
Expression of *PRPF3* RNA and protein in mouse tissues. (a) *PRPF3* mRNA levels in mouse tissues. *PRPF3* mRNA at indicated time points detected with real-time RT-qPCR. Total RNA was isolated from liver, lung, kidney heart, spleen, brain, retina, muscle, and intestine (n = 3 groups). The result was normalized with 18S. * = p<0.05, ** = p<0.01 retina compare to brain. (b) Prpf3 protein in mouse tissues. Prpf3 protein detected with Western blots at indicated time points. E15.5d, embryonic day 15.5; 5d, postnatal day 5; 1 m, one month; 4 m, four months; 1 y, one year.

Given its role in splicing, *PRPF3* is assumed to be ubiquitously expressed. Even if this is the case, the levels of its expression might vary in different tissues and developmental stages. If more *PRPF3* function is required in retinal cells, we would expect a higher level of *PRPF3* expression in the retina. To examine this idea, we determined mRNA levels of *PRPF3* in mouse tissues at indicated time points via quantitative RT-PCR. At embryonic day 15.5 (E15.5d) and postnatal day 5 (5d), there was no major difference in the levels of *PRPF3* mRNA among mouse tissues (liver, lung, kidney, heart, spleen, brain, retina, muscle and intestine). However, the levels of retinal expression were higher in one-month (1 m) and four-month (4 m) old mice compared to other tissues. A high level of *PRPF3* mRNA in the retina is sustained in one-year old mice compared to another neural tissue, brain (Fig. 1a and Table 1) (p<0.05).

**Table 1:** Summary of mRNA levels of splicing factor genes in retina tissue.

gene	E15.5d	5 days	1 month	4 month	1 year
*PRPF3*	**-**	**-**	**++**	**+**	**+**
*PRPF31*	**-**	**-**	**-**	**++**	**++**
*PRPC8*	**-**	**-**	**+**	**+**	**-**
*PRPF4*	**-**	**-**	**-**	**-**	**-**
U1-70K	**-**	**-**	**-**	**-**	**-**
U2-SF3a	**-**	**-**	**-**	**-**	**-**

+: p<0.05; ++: p<0.01 (mRNA levels in retina is significantly higher than that in brain); -: no difference.

To investigate whether Prpf3 protein expression correlates with mRNA expression, we examined the levels of Prpf3 protein in different mouse tissues at different stages of development using immunoblotting assays (Fig. 1b). As expected, mouse Prpf3 protein was expressed in all tissues analyzed. Since different organs have distinct cell types that express different amounts of structural proteins, a particular structural protein, such as β-actin, cannot be used as an internal standard because its relative abundance in various tissues is different. In order to normalize the levels of the mouse Prpf3 protein, we measured the total protein concentration from each sample and loaded equal amounts of protein (100 µg) on SDS-PAGE followed by either immunoblotting or Coomassie Brilliant Blue staining. We found that the level of Prpf3 protein expression was similar among different tissues in 5 day and 1 month old mice. However, the level of Prpf3 protein was higher in the retina than in any of the other tissues examined after the mice reached adulthood (around 4 months). These protein expression results are consistent with the RNA expression data. In older mice, retinal expression of Prpf3 protein decreased to levels similar to that found in brain tissue.

### Localization of *PRPF3* mRNA in different types of mouse retinal cells

**Figure 2:**
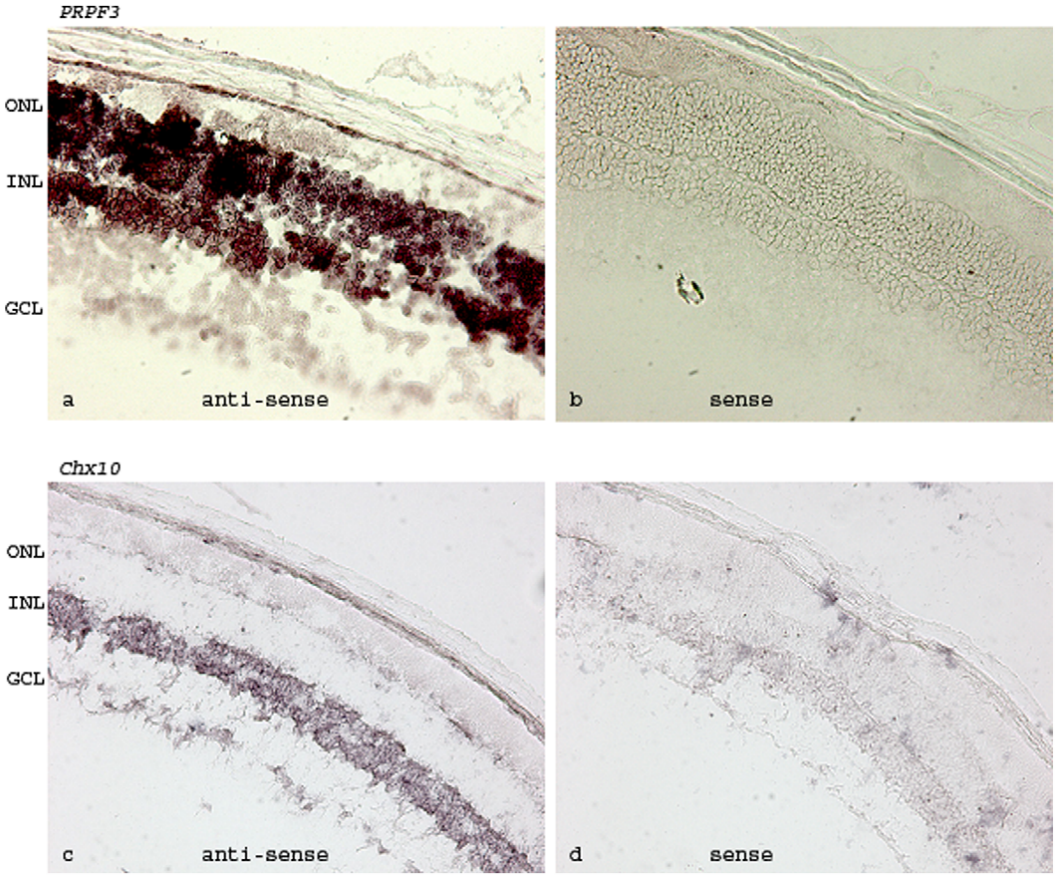
Expression of *PRPF3* gene in mouse retina. *In situ* hybridization with an antisense (a) or sense (b) probe against *PRPF3*. (c and d) *In situ* hybridization with an antisense (c) or sense (d) probe against *Chx10* gene used as a control for *in situ* hybridization. *Chx10* expression is located in the inner nuclear layer. ONL: outer nuclear layer, INL: inner nuclear layer, GCL: ganglion cell layer.

Strong retinal expression of *PRPF3* does not necessarily mean a high level of expression in photoreceptor cells since the retina contains several different cell types. To examine the *PRPF3* mRNA distribution in different retinal cells, we performed *in situ* hybridization analysis using 4-month old mice (Fig. 2). The expression of *PRPF3* mRNA was detected in all cellular layers of the retina and a high level of *PRPF3* mRNA was observed in photoreceptor cells. *In situ* hybridization signals were specific to *PRPF3* as they were not detectable with the sense probe used as a control**.** To further validate the *in situ* conditions, we performed *in situ* hybridization using a probe against *Chx10* gene that is primarily expressed in the inner nuclear layer (INL) of the mature mouse retina [23]. Indeed, we observed *Chx10* signals only in this layer of the retina while no signal was detected using the *Chx10* sense probe.

### Developmental expression of splicing factors *PRPF31*, *PRPC8*, *PRPF4*, and U1-, U2-RNP-specific splicing factors in mouse tissues

**Figure 3:**
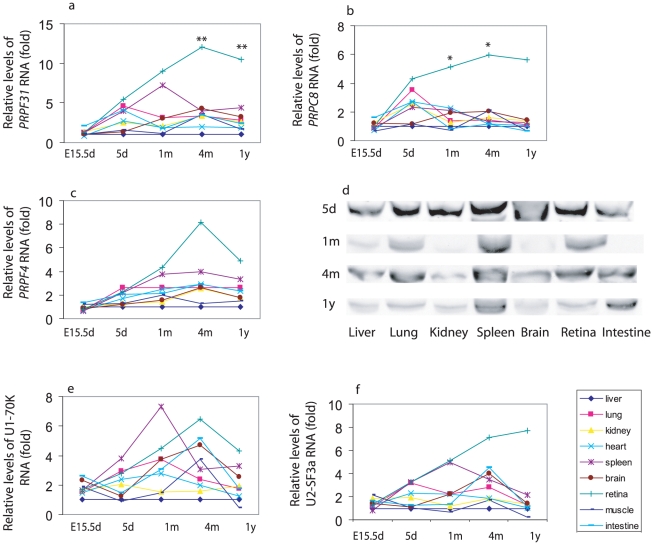
Expression of *PRPF31*, *PRPC8*, *PRPF4*, U1-70K and U2 SF3a genes RNA in mouse tissues. (a) *PRPF31* mRNA levels in mouse tissues. *PRPF31* mRNA detected by real-time RT-qPCR. ** = p<0.01 retina compare to brain. (b) *PRPC8* mRNA levels in mouse tissues. * = p<0.05, retina compare to brain. (c) *PRPF4* mRNA levels in mouse tissues. (d) Prpf4 protein in mouse tissues. Prpf4 protein was detected with Western blots at indicated time points. (e) U1-70K gene mRNA levels in mouse tissues. (f) U2 SF3a gene mRNA levels in mouse tissues. For abbreviations, see the legend in Fig. 1.

Prpf3 is part of U4/U6-U5 tri-snRNP, whereas Prpf31 binds to the U4 snRNP and Prpc8 interacts with U6 RNA. All the three splicing genes are ubiquitously expressed and associated with autosomal dominant RP (RP18, RP11 and RP13, respectively). As *PRPF3* mRNA was highly expressed in the adult mouse retina, we decided to analyze the developmental expression of the other two U4/U6-U5 associated genes (*PRPF31* and *PRPC8)*. Interestingly, the mRNA levels of *PRPF31 and PRPC8* are very similar to those of *PRPF3*. In E15.5d and 5d mice, the mRNA levels of *PRPF31* and *PRPC8* were not significantly different among nine different tissues (Fig. 3a, b). Likewise, the *PRPF31* and *PRPC8* mRNA levels are significantly higher in the retina than in all other tissues in four month old mice. The levels of *PRPF31* and *PRPC8* mRNA in the brain are 34–36% of that detected in the retina (Table 1).

As the human *PRPF3* gene product, Hprp3p, is stably and directly associated with the *PRPF4* gene product Hprp4p within the U4/U6 snRNP [6], [24], we examined whether the temporal and spatial expression patterns of the *PRPF4* gene in mice are similar to those observed for *PRPF3*. Unlike *PRPF3*, the levels of *PRPF4* mRNA and protein in the retina were not higher than those in all other tissues (Fig. 3c, d).

In additional to the U4/U6-U5 associated genes encoding splicing factor Prpf3, Prpf31, Prrpc8 and Prpf4, we also determined the mRNA levels of genes encoding the U1-70K protein and U2-SF3a in mouse tissues (Fig. 3e, f). U1-70K is a U1 snRNAP-associated protein while SF3a is component of the U2 snRNP and required for complex A assembly [25]. We found that the U1-70K protein and SF3a genes were consistently expressed at low levels in all mouse tissues at various developmental stages. There was no significantly difference in the mRNA levels of U1-70K and SF3a among the tissues analyzed.

### Levels of snRNAs, U1, U2, U4, U5, and U6 in mouse tissues

**Figure 4:**
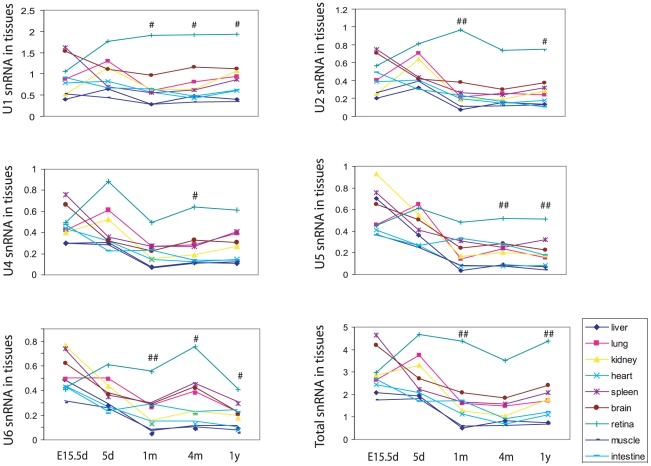
Expression of snRNAs in mouse tissues. U1, U2, U4, U5, U6, and the total snRNAs were detected by Northern blotting with snRNA-specific probes as described in Materials and Methods and Figure S1. The RNA signals detected with the probes were normalized to 5S rRNA. * p<0.05, ** p<0.01 retina compare to brain. For abbreviations, see the legend in Fig. 1.

The snRNAs U1, U2, U4, U5, and U6 are major components of spliceosomes and these highly conserved snRNAs play a central role in all aspects of the splicing reaction. Previous studies have shown that Hprp3p is a double stranded RNA binding protein that specifically contacts U4/U6 snRNAs through both its central and C-terminal regions [6], [26]. Since *PRPF3* is highly expressed, it is possible that snRNAs may also display similar expression patterns. With that in mind, we analyzed the temporal and spatial expression patterns of mouse snRNAs using Northern blotting (Figure S1). We found that there was no significant difference in the levels of the five snRNAs in 15.5d embryos and postnatal day 5 mouse tissues between the retina and all other tissues (Fig. 4). However, the levels of all snRNAs are much higher in the retina of one-month, four-month and one-year old mice than in other tissues. The levels of U1, U4, U5 and U6 in the retina of four-month mice were significantly higher than in the brain.

### RP18 mutation of *PRPF3* does not affect retina-specific gene expression

Currently, there is no direct evidence indicating that mutations of *PRPF3* affect retina-specific gene splicing in RP18 patients. In a cultured human retinal epithelial cell line, ARPE-19, no splicing defect was observed when the endogenous Hprp3p was replaced by the RP18 mutant protein via a helper-dependent adenoviral vector that simultaneously knocks down the endogenous Hprp3p and expresses a copy of the RP18 mutant *PRPF3* [27]. However, it is not known whether RP18 mutation affects RNA splicing in photoreceptor cells. To address this question, we isolated total RNA from the eyeballs of RP18 knockin mic[28], [29] and measured mRNA levels of several retina-specific genes (including photoreceptor-specific genes) since a decrease in levels of mRNA may indicate a defect in RNA splicing. Compared to the wild-type mice, we did not find a difference in mRNA levels of RHO (phototransduction gene rhodopsin), NRL (transcription factor neural retina leucine zipper), RP1 (retinitis pigmentosa 1 gene) and RDS (photoreceptor structure gene retina degeneration slow). For comparison, we also examined the mRNA levels of a house keeping gene, β-2-microglobulin and a single exon gene Histone 4. These data further demonstrate that RP18 mutation does not cause a major defect in splicing function, nor does it affect the splicing of retina-specific and house-keeping genes (Table 2). To further confirm this, we examined the effect of the RP18 mutation on rhodopsin protein expression in mouse retinal primary culture cells. We transduced the primary cells with a helper-dependent Adenovrial (HD-Ad) vector expressing shRNAs targeting the endogenous *PRPF3* and simultaneously expressing a copy of the wild type or RP18 mutant *PRPF3* [30]. Double immunostaining was performed to detect exogenous Hprp3p tagged with HA and rhodopsin in photoreceptor cells. We did not detect a noticeable difference in rhodopsin protein expression in photoreceptor cells between wild-type and RP18 mutant Hprp3p (Figure S2) suggesting that RP18 mutation of *PRPF3* does not affect rhodopsin gene expression in mouse photoreceptor cells over a short period of time.

**Table 2:** mRNA levels of retina specific, splicing factor, and house keeping genes in the eye of WT and RP18 mice.

	*PRPF3*-WT	RP18m
**Retina specific genes:**		
Phototransduction: *RHO*	1	1.131
Transcription Factor: *NRL*	1	1.001
Transport Gene: *RP1*	1	0.998
Photoreceptor Structure Gene: *RDS*	1	1.011
**pre-mRNA Splicing Factor Genes:**		
*PRPF3*	1	1.620*
*PRPF31*	1	1.056
*PRPC8*	1	1.041
**Single Exon Gene:**		
*H4d*	1	0.951
**House Keeping Gene:**		
*Beta 2M*	1	1.158

mRNA levels (fold) of retina specific, splicing factor, and house keeping genes in the eye of WT and RP18 (*PRPF3* T494M/T494M) knockin mice, detected by real-time RT-qPCR, n = 5.

*: p<0.05, comparing to WT.

*Rho*:Rhodopsin; *NRL*: neural retina leucine zipper; *RP1*: retinitis pigmentosa 1; *RDS*: retina degeneration slow; *H4d*: Histone cluster 1, H4d; *Beta 2M*: beta 2-Microglobulin.

### Cellular localization of wild-type and RP18 mutant protein in cultured human retinal epithelial cells

**Figure 5:**
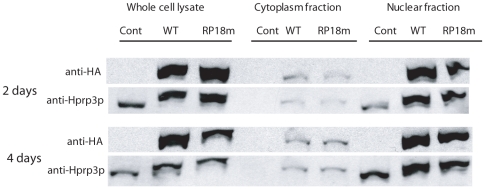
Localization and solubility of WT and RP18 mutant protein. ARPE19 cells were transduced with HD-Ad-F3iplus and HD-Ad-F3iT494M vectors, which knock down the endogenous *PRPF3* expression and express an exogenous copy of wild-type or T494 M mutant *PRPF3*, respectively. Control cells were transduced with the HD-Ad empty vector. Proteins in the transduced cells were isolated and fractionated. The exogenous and the endogenous Hprp3p proteins were detected with an anti-Hprp3p antibody. The anti-HA antibody was used to detect the exogenous Hprp3p protein only.

Mutated proteins can change their properties, such as solubility or cellular distribution, which may lead to pathological symptoms [31], [32]. Therefore, we investigated whether RP18 mutation altered the cellular localization of Hprp3p in human retinal epithelial cell lines. Currently there is no *PRPF3*-deficient mammalian cell line for studying protein localization. However, the effect of a dominant mutation can be monitored in cells transduced with a mutant gene while the endogenous gene product is knocked down by RNA interference [27]. Small hairpin RNA was used to specifically silence the endogenous *PRPF3* protein while simultaneously expressing HA-tagged human wild-type or Thr494Met *PRPF3*, using a viral vector HD-Ad-F3iplus or HD-Ad-F3iT494M, respectively [27], [30]. We found that more than 90% of the endogenous Hprp3p was knocked down by HD-Ad-F3iplus or HD-Ad-F3iT494M at two and four days compared to the empty vector control. The majority of both exogenous wild-type and mutant Hprp3p proteins was located in nucleus (Fig. 5). The total levels of the wild-type and mutant Hprp3p protein expressed in ARP19 cells were similar. The protein from nuclear fraction was approximately equal to that from the whole cell lysate in both wild type and mutant human *PRPF3* transduced cells as determined by immunoblotting. This indicated that the majority of the expressed wild-type and Thr494Met mutant proteins was soluble and located in the nucleus. Interestingly, we detected the presence of Hprp3p in the cytosolic fraction from cells transduced with viral vectors expressing either wild-type or Thr494Met *PRPF3* at two and four days. We did not observe any Hprp3p signal in the cytosolic fraction from control cells (transduced with the empty vector) on the immunoblot, even with longer exposure (data not shown). The presence of Hprp3p in the cytoplasmic fraction was unlikely caused by leakage from the nuclear fraction since nuclear-localized RNA polymerase II was not detected in the same cytoplasmic fraction. This may have been caused by overexpression of the exogenous Hprp3 protein. There was no difference in the levels of cytosolic proteins between the wild-type and Thr494Met Hprp3p.

## Discussion

How mutations in the three genes encoding U4/U6-U5 tri-snRNP associated splicing factors cause adRP is still an intriguing question. The mutant proteins could theoretically confer a true dominant phenotype by gaining a function that produces a detrimental effect on photoreceptor cells (such as interrupting a cell-specific interaction or generating a cytotoxic product). Alternatively, the dominant phenotype may be due to haploinsufficiency. Therefore, analyses of the expression pattern of these genes will help us gain insight into the RP disease mechanisms assuming the expression patterns of these splicing factors in mice and humans are similar.

We performed, for the first time, extensive temporal and spatial expression analyses of RP-associated splicing factors, Prpf3, Prpf31 and Prpc8, and other splicing factors, Prpf4, U1-70K protein and SF3a as well as snRNAs in mice in order to gain information on tissue specific expression and developmental regulation of the splicing machinery. We found that only RP-associated splicing factor genes *PRPF3, PRPF31* and *PRPC8* display a high level of expression in the retina compared to other tissues, in postnatal mice. Our studies show that the temporal pattern of this high level of retinal expression correlates with the visual activity during mouse development. For example, in late fetal and neonatal stages, the level of retinal expression of these splicing factors was comparable to other tissues; this correlates with the lack of visual activity in these developmental stages. Our results indicate that postnatal retinas require higher levels of some splicing components than other metabolically active tissues for daily normal visual function. We also found that snRNAs are also highly expressed in retinas in postnatal mice (Fig. 4). With visual function developing, the levels of snRNAs become higher than any other tissues. The difference in cell-specific basal levels of RP-associated splicing factors under normal physiological condition may contribute to the difference in sensitivity of different cell types to these splicing factor mutations.

Currently there is no direct evidence suggesting unique functions for RP-associated splicing factors or showing a high level of splicing activity in retinal cells. However, it is well known that photoreceptors are highly specialized, non-dividing cells with high oxygen consumption and an unusually high metabolic rate [33], [34]. Rod and cone photoreceptors turn over their outer segments every 10 days. The constant renewal of membranes in these cells require the active biosynthesis of phospholipids and neutral lipids, and a high level of expression of both retina-specific and housekeeping genes, a dynamic transport system and a huge energy supply. These unique properties make the photoreceptor cells sensitive to a variety of genetic and environmental insults. The high demand components for pre-mRNA processes and oxygenation changes may contribute to the selective vulnerability of retina in retinitis pigmentosa [35].

The clinical data from RP11 individuals show that the phenotype of RP11 correlates with the reduced expression level of *PRPF31* mRNA in patients' lymphoblasts [36]. These clinical data are consistent with the results of our gene expression analysis in mice, suggesting that expression levels of RP-associated genes are important for vision function. The loss of one functional copy of these essential splicing genes, without compensating expression, may lead to insufficient function in supporting photoreceptors especially because of their high demand for mRNA expression and protein synthesis. Interestingly from the clinical data, the mRNA level in RP11 asymptomatic carriers was closer to that in control individuals than to that in symptomatic patients. The level of *PRPF31* mRNA and protein in lymphoblasts from affected patients was significantly lower than that from asymptomatic carriers with same mutations [36], [37]. This also implies a compensatory regulation in asymptomatic carriers with RP11 mutation. It is possible that asymptomatic individuals have a different wild-type *PRPF31* allele whose expression may be elevated to compensate for the functional loss of the mutant allele.

However, genetic studies in mice showed a different picture. Heterozygous mice with one copy of the *PRPF3* or *PRPF31* genes knocked out do not cause retinal degeneration [38], [39]. It is possible that the life span of mice is too limited to develop the RP symptom or the artificial light conditions in animal facilities are not appropriate for producing the RP symptom. However, we cannot rule out the possibility that RP-associated splicing factors may possess unknown functions required only for processing some retina-specific transcripts and thus, a decrease in their expression or in their function due to genetic mutations may lead to RP.

In summary, our study showed that high levels of RP-associated splicing factors and snRNAs in mouse retinal cells correlate with the developmental regulation of the visual function. The high level of expression of these genes in retina may partially explain the selective vulnerability of retinal cells in the case of RP18, 11 and 13. Although a minor splicing functional defect can be tolerated by mice which have a shorter life span, it may render an accumulative effect on retinal degeneration in humans.

## Materials and Methods

### Animal and tissue sample preparation

Wild type CD-1 female mice were obtained from Charles River Laboratories and maintained as per guidelines of Sick Kids animal facilities. The RP18 (*Prpf3*-T494M/T494M) knockin mice were described by Graziotto *et al*.[28]. All animal studies were reviewed and approved by the Sick Kids institutional committee for humane use of laboratory animals. Mouse tissues were collected from different organs at time points as indicated in figure legends and pooled together. Organs from five mice were pooled together for each group and three groups were included at each time point. Retinal material was dissected from eyeballs by circumferential section of the cornea and removal of the anterior chamber as well as major blood vessels. Mouse tissues were homogenized in Brij buffer (1M Tris-HCl pH 7.5, 0.5M EDTE, 5M NaCl, 10% Brij 96 and 10% NP40) with protein inhibitors (complete protease inhibitor cocktail tablets, Roche) for protein sample preparation or in Trizol solution for RNA isolation.

### Western blot

Protein concentration of tissue lysates was measured, and equal amounts of protein (100 µg) for each sample were loaded on a SDS-PAGE followed by either immunoblotting detection or Coomassie Brilliant Blue staining. Proteins in the SDS-PAGE gel were transferred electrophoretically to nitrcellulose membrane and the membrane was blocked in TBST containing 5% nonfat dry milk and 2% BSA for 2 hour at room temperature. Primary antibodies against Prpf3 and Prpf4 were described previously [4]. HRP–conjugated anti–rabbit secondary antibodies (Vector Laboratories, Burlingame, CA) were used in conjunction with ECL reagent (Amersham Pharmacia Biotech, Canada). The proteins on the blot were detected by X-ray film. Coomassie Brilliant Blue staining of a separate gel was used to confirm that same amount of proteins was loaded in each lane.

### *In situ* hybridization

*In situ* hybridization was performed on mouse retinas with an antisense RNA probe of *Prpf3* with the sense probe as a control using a protocol as described previously [40]. Briefly, digoxygenin (DIG)-labeled sense and antisense probes for *Prpf3* and Chx10 genes were prepared from linearized *Prpf3* cDNA plasmid or Chx10 cDNA plasmid in *in vitro* transcription using a DIG–RNA labeling kit (Roche). Retinal sections were fixed in 4% paraformaldehyde in PBS, pH 7.6. After treating with triethanolamine and acetic anhydride, the slides were prehybridized for 2 h at 60°C in a hybridization solution (50% formamide, 5xSSC, 5xDenhardt's solution, 0.25 mg/ml yeast tRNA, and 0.5 mg/ml heparin) and then hybridized in the probe-containing hybridization solution overnight at 60°. After washing in SSC buffers (2× to 0.1× SSC), slides were incubated with anti-digoxygenin–alkaline phosphatase–Fab fragment in 10% goat serum in TBST overnight at 4°C. After washing, the color was developed with alkaline phosphatase substrates (nitroblue-tetrazolium-chloride/5-bromo-4-chloro-3-indolyl- phosphate) in the dark. Slides were mounted and photographed.

### RNA isolation and real-time q-PCR

Total RNA was extracted using the TRIzol reagent (Invitrogen) according to manufacturer's instructions. For qRT–PCR, contaminating DNA was removed using RNase-free DNase I. One ug of RNA was reverse-transcribed using the SuperScript II (Invitrogen); real-time PCR was performed as described [41]. For relative quantification, PCR signals were compared among tissues after normalization to 18S rRNA (Ribosomal RNA Control Reagents, ABI). Fold change was calculated according to Livak and Schmittgen [28] and normalized to liver tissue. The primers are listed in Table 3.

**Table 3:** Primers used for real-time RT-qPCR.

Gene	FORWARD	REVERSE	Accession No.	Product length
*PRPF3*	GGTGGGACTCGTACATCATAC	GGGTAACTGGTGTATCATTGTCA	NM_027541	141 bp
*PRP4*	CCTCCTCCACGACAACCAAA	CCCAAAATCCCAGACTCTCC	NM_027297	136 bp
*PRPF31*	GAGAGCACAGAAGGGAAGG	GGGCTTCACCTGCTTCACG	NM_027328	99 bp
*PRPC8*	GTGGATGCTCAGAAGGAAGACA	GAGGACTGCATGAGGCATGTA	NM_138659	141 bp
*U1-70K*	CGGAGAGAGTTTGAGGTGTATG	GTGCTTGTAAGCGGAGTGCAT	NM_009224	133 bp
*U2-SF3a*	GGAATTTGAGGAGCTGCTGAAG	GGTAGCAGTCATGGAGATCAAG	NM_029157	119 bp
*Rho*	GCCACACTTGGAGGTGAAATC	CCACACCCATGATAGCGTGAT	NM_145383	125 bp
*NRL*	CTGTGCCTTTCTGGTTCTGACA	GGACTGAGCAGAGAGAGGT	NM_008736	83 bp
*RP1*	GCAGGTTGCATATCCAGTGAC	GGAGAAACTAGTAGAAGGTGTGT	BC_0996607	123 bp
*RDS*	GGAGGTCAAAGATCGCATCAAG	GGTGAGCTGGTACTGGATACA	NM_008938	115 bp
*H4d*	CATCACCAAGCCCGCCATC	GGAACACCTTCAGCACACCA	NM_175654	101 bp
*Beta 2M*	GGTCGCTTCAGTCGTCAGCAT	GCAGTTCAGTATGTTCGGCTTC	NM_009735	154 bp

*Rho*:Rhodopsin; *NRL*: neural retina leucine zipper; *RP1*: retinitis pigmentosa 1; *RDS*: retina degeneration slow; *H4d*: Histone cluster 1, H4d; *Beta 2M*: beta 2-Microglobulin.

### Northern blot for detecting snRNAs

For Northern blotting detection of snRNAs in mouse tissues, equal amounts of total tissue RNA (2 ug) were separated on a 10% polyacrylamide-urea (8 M) gel, transferred to nylon membrane (Roche Diagnostics) [42], and UV cross-linked. The membrane-bond RNA was hybridized using ExpressHybTM (BD Biosciences, Mississauga, ON, Canada) to ^32^P-labled oligonucleotide probes complementary to U1, U2, U4, U5 and U6 snRNAs at 42°C. The snRNA sequences probed were as follows: U1 (1-79), U2 (8-87), U4 (1-85), U5 (18-88), U6 (32-99) [42], [43], in Figure S3. Blots were quantified using the PhosphorImager and ImageQuant software, and normalized with 5S rRNA.

### Preparation of HD-Ad vectors

HD-Ad-F3iplus and HD-Ad-F3iT494M were described previously [30]. Both HD-Ad vectors express shRNAs that target the endogenous PRPF3 mRNA as well as a copy of HA-tagged PRPF3 cDNA driven by the human UbC promoter. The HD-Ad-F3iplus expresses the wild-type human *PRPF3* while HD-Ad-F3iT494M expresses one with the RP mutation (T494M). The viral vector were prepared as described [27], [30].

### Cell culture and protein fractionation

ARPE19 cells were cultured in DMEM-F12, supplemented with 10% fetal bovine serum (FBS) (Invitrogen Canada Inc., Burlington, ON, Canada). Cells were transduced at 70% confluency with HD-Ad-F3iplus or HD-Ad-F3iT494M, or empty HD-Ad vectors (2500 particles/cell) under serum-free conditions for 2 h, followed by the addition of media to a final concentration of 10% FBS. Cells were harvested by scraping 48 h or 96 h post transduction. The cell pellet from one each 10 cm dish was resuspended in 1 ml buffer A (10 mM HEPES pH 7.9, 1.5 mM MgCl_2_, 10 m M KCl, 0.5 mM DTT, 0.5 mM PMSF, 0.25 mM benzamidine) and incubated on ice. The cells were re-pelleted and then resuspended in 300 µl buffer A. For each preparation a 100 µl aliquot was removed for analysis of whole cell proteins. NaCl was added to this aliquot to a concentration of 1 M to lyse cell membranes and release proteins. To the remaining 200 µl of cell suspension, the detergent Nonidet P-40 was added to a concentration of 0.2% and the mixture was incubated on ice until all cell membranes were lysed. The suspension was centrifuged to isolate the supernatant containing soluble cytosolic proteins. The pellet containing nuclei and insoluble components was resuspended in 200 µl buffer B (5 mM HEPES pH 7.9, 1.5 mM MgCl_2_, 0.2 mM EDTA, 0.5 mM DTT, 0.5 mM PMSF, 0.25 mM benzamidine) and NaCl was added to a concentration of 1 M. The suspension was then centrifuged and the supernatant containing soluble nuclear proteins was preserved [31]. The protein concentration of all extracts was determined using an ABC protein assay kit (Pierce) with BSA as a standard. Extracts from cells transduced empty HD-Ad vector were used a control for protein fractionation and Western-blotting.

### Primary cell culture and immunofluorescent staining

Retinas were isolated in fresh Ringer's solution and chopped into small fragments, washed in Ringer's solution without Ca_2_+ and Mg_2_+, supplemented with 0.1 mM EDTA, and incubated in 0.5 ml 0.2% activated papain (Sigma) in the same buffer at 37°C for 20 minutes. The tissue was dissociated by adding 1 mL of DMEM/F12 and 15 µl of 10 mg/mL DNase I, and repeated gentle triturating. Cells were seeded in DMEM/F12 (Gibco), supplemented with 10% fetal calf serum (FCS; Gibco) and penicillin-streptomycin (10 IU/L), into 6-well tissue culture plates containing coverslips previously coated with poly-L-lysine (2 mg/cm^2^ for 2 hours) followed by laminin (1 mg/cm 15 minuts; both from Sigma–Aldrich). Cells were incubated at 37°C in a humidified atmosphere of 5% CO2 [44] and were transduced with HD-Ad-F3iplus and HD-Ad-F3iT494M.

Double immunofluorescent labeling was performed on the primary culture of mouse retinal cells one week after transduction with HD-Ad-F3iplus and HD-Ad-F3iT494M vectors. The first primary antibody against-HA-tag was used to detect exogenous Prpf3 protein with anti-mouse IgG fluorescence (green). The endogenous mouse rhodopsin protein was detected by the primary antibody against-rhodopsin with anti-rabbit IgG conjugated-Cy3 (red) as the secondary antibody. Confocal microscopy was performed using a Zeiss inverted spinning disk confocal microscope and analyzed with Volocity software (Perkin Elmer Inc., Waltham, MA).

### Statistical analysis

Student's t-test or one-way ANOVA followed by Holm's multiple comparison was used to compare data pairs or sets, respectively. Data are presented as mean ± SEM. p<0.05 was considered statistically significant.

## Supporting Information

Figure S1 **A representative Northern blot of snRNAs of mouse tissues.** Northern-blotting was performed as described in Methods and Materials. The RNA bands are designated on the right side of the bottom panel. Abbreviations: E15.5d, embryonic day 15.5; 1 m, one month; 4 m,four months, 1 y, one year; U1, U1 snRNA; U2, U2 snRNA; U4, U4 snRNA; U5, U5 snRNA; U6, U6 snRNA, 5S, 5s rRNA; li, liver; lu, lung; ki, kidney; he, heart; sp, spleen; br, brain; re, retina; mu, muscle; int, intestine.
(TIF)

Figure S2 **Expression of wild-type and RP18 mutant human** ***PRPF3*** **in mouse photoreceptor cells.** Mouse retinal primary cells were prepared and transduced with viral vectors, HD-Ad-F3iplus and HD-Ad-F3iT494M (See methods). The exogenously expressed HA-tagged human Hprp3p protein and mouse endogenous rhodopsin protein were detected with anti-HA (green) or anti-rhodopsin antibody (red), respectively, one week following transduction by double immunofluorescent staining. DAPI staining was used to show the nuclei.
(TIF)

Figure S3 **Oligonucleotides used as probes for snRNA Northern Blot.** For oligo probes used for snRNAs, the nucleotide positions are provided following the names of the oligos. These oligo probes were previously used [Cooper M, Johnston LH, Beggs JD (1995) Identification and characterization of Uss1p (Sdb23p): a novel U6 snRNA-associated protein with significant similarity to core proteins of small nuclear ribonucleoproteins. Embo J 14: 2066-2075; Patel AA, Steitz JA (2003) Splicing double: insights from the second spliceosome. Nat Rev Mol Cell Biol 4: 960-970].
(EPS)
