# Supplementary figures and images for: Temporal and Tissue Specific Regulation of RP-Associated Splicing Factor Genes PRPF3, PRPF31 and PRPC8—Implications in the Pathogenesis of RP
Source: PLoS One. 2011 Jan 19;6(1):e15860. doi: 10.1371/journal.pone.0015860 (PMC3023711; doi:10.1371/journal.pone.0015860)

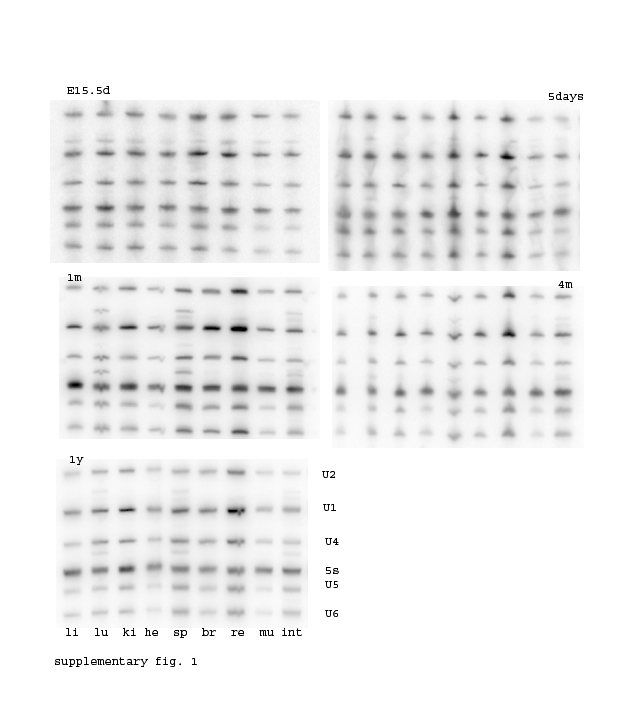

Supplement: Figure S1 — A representative Northern blot of snRNAs of mouse tissues. Northern-blotting was performed as described in Methods and Materials. The RNA bands are designated on the right side of the bottom panel. Abbreviations: E15.5d, embryonic day 15.5; 1 m, one month; 4 m,four months, 1 y, one year; U1, U1 snRNA; U2, U2 snRNA; U4, U4 snRNA; U5, U5 snRNA; U6, U6 snRNA, 5S, 5s rRNA; li, liver; lu, lung; ki, kidney; he, heart; sp, spleen; br, brain; re, retina; mu, muscle; int, intestine. (TIF) [file pone.0015860.s001.tif]

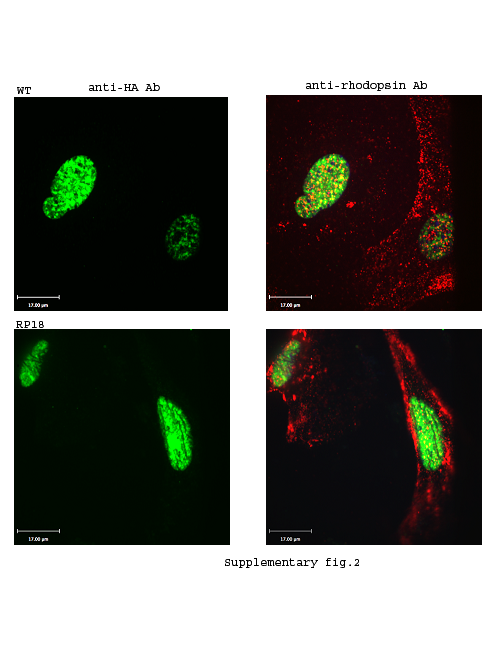

Supplement: Figure S2 — Expression of wild-type and RP18 mutant human PRPF3 in mouse photoreceptor cells. Mouse retinal primary cells were prepared and transduced with viral vectors, HD-Ad-F3iplus and HD-Ad-F3iT494M (See methods). The exogenously expressed HA-tagged human Hprp3p protein and mouse endogenous rhodopsin protein were detected with anti-HA (green) or anti-rhodopsin antibody (red), respectively, one week following transduction by double immunofluorescent staining. DAPI staining was used to show the nuclei. (TIF) [file pone.0015860.s002.tif]
